# State anxiety influences P300 and P600 event-related potentials over parietal regions in the hollow-mask illusion experiment

**DOI:** 10.1017/pen.2020.16

**Published:** 2021-02-16

**Authors:** Vasileios Ioakeimidis, Nareg Khachatoorian, Corinna Haenschel, Thomas A. Papathomas, Attila Farkas, Marinos Kyriakopoulos, Danai Dima

**Affiliations:** 1 Department of Psychology, School of Arts and Social Sciences, City, University of London, London, UK; 2 Center for Cognitive Science, Rutgers University, Piscataway, NJ, USA; 3 Department of Biomedical Engineering, Rutgers University, Piscataway, NJ, USA; 4 National and Specialist Acorn Lodge Inpatient Children Unit, South London and Maudsley NHS Foundation Trust, London, UK; 5 Department of Child and Adolescent Psychiatry, Institute of Psychiatry, Psychology and Neuroscience, King’s College London, London, UK; 6 Department of Neuroimaging, Institute of Psychiatry, Psychology and Neuroscience, King’s College London, London, UK

**Keywords:** Hollow-mask illusion, EEG, P300, P600, Neuroticism, Anxiety

## Abstract

The hollow-mask illusion is an optical illusion where a concave face is perceived as convex. It has been demonstrated that individuals with schizophrenia and anxiety are less susceptible to the illusion than controls. Previous research has shown that the P300 and P600 event-related potentials (ERPs) are affected in individuals with schizophrenia. Here, we examined whether individual differences in neuroticism and anxiety scores, traits that have been suggested to be risk factors for schizophrenia and anxiety disorders, affect ERPs of healthy participants while they view concave faces. Our results confirm that the participants were susceptible to the illusion, misperceiving concave faces as convex. We additionally demonstrate significant interactions of the concave condition with state anxiety in central and parietal electrodes for P300 and parietal areas for P600, but not with neuroticism and trait anxiety. The state anxiety interactions were driven by low-state anxiety participants showing lower amplitudes for concave faces compared to convex. The P300 and P600 amplitudes were smaller when a concave face activated a convex face memory representation, since the stimulus did not match the active representation. The opposite pattern was evident in high-state anxiety participants in regard to state anxiety interaction and the hollow-mask illusion, demonstrating larger P300 and P600 amplitudes to concave faces suggesting impaired late information processing in this group. This could be explained by impaired allocation of attentional resources in high-state anxiety leading to hyperarousal to concave faces that are unexpected mismatches to standard memory representations, as opposed to expected convex faces.

Visual illusions are primarily, for research, a great tool to understand human perception (Carbon, 2014) and they occur when the subjective percept does not match the real physical properties of the observed object. This mismatch can be a result of stimulus-driven assumptions made by the visual system and other times they constitute an active recalibration of higher-level cognitive areas (Eagleman, 2001). They can be distinguished in two categories based on the brain networks which contribute to the illusory percept; illusions resulting from bottom–up signals are called physiological or low-level, whereas those occurring from top–down regulatory activity are cognitive illusions (Dima, Dillo, Bonnemann, Emrich & Dietrich, 2011; King, Hodgekins, Chouinard, Chouinard & Sperandio, 2017).

An interesting class of cognitive illusions is the binocular depth-inversion illusions that result in objects perceived in reverse depth, with distant points perceived to be closer than near points; thus, concavities are perceived as convexities and vice versa. The best-known binocular depth-inversion illusion is the hollow-mask illusion. One way to experience it is to swap the images of the left and right eyes of a stereoscopic pair; despite the strong stereoscopic cues that signal a concave mask, viewers report perceiving a normal convex face (Farkas, Papathomas, Silverstein, Kourtev & Papayanopoulos, 2016; Georgeson, 1979; Van den Enden & Spekreijse, 1989). A similar experience can be perceived by using a physical hollow mask, prompting a misperception of the concave 3D surface as convex, despite visual depth cues suggesting the opposite (concave when a face is perceived as 3D going inwards and convex when it was going outwards, like a normal face) (Gregory, 1973; Hill & Bruce, 1993; Hill & Johnston, 2007; Papathomas & Bono, 2004). Healthy controls from 6 month of age (Corrow, Granrud, Mathison & Yonas, 2011) throughout adulthood incorrectly perceive concave faces as convex. Individuals with schizophrenia perceive the illusion to a much lesser degree than controls; instead, they have a veridical perception of the truthful concavity of the mask (Schneider et al., 2002). Schneider et al. (2002) argued that this incongruence is the result of disturbed top–down processes in individuals with schizophrenia, which can be reversed after the course of antipsychotic medication. This discrepancy in schizophrenia has been shown to be due to strengthened bottom–up and weakened top–down processing that allows schizophrenia patients to interpret the sensory cues of a hollow face that deviate from stored knowledge of faces being convex as concave (Dima, Dietrich, Dillo & Emrich, 2010; Dima et al., 2011, 2009). Apart from schizophrenic patients, individuals with other psychosis-prone states are also less likely to perceive the hollow-mask illusion, such as cannabis users (Leweke, Schneider, Radwan, Schmidt & Emrich, 2000; Semple, Ramsden & McIntosh, 2003), alcohol withdrawal (Schneider et al., 1996), sleep deprivation (Sternemann et al., 1997), youth at ultra-high risk for psychosis (Gupta et al., 2016), and anxiety patients (Passie et al., 2013). Therefore, disposition of veridical perception of concaveness in faces in the psychotic and pro-psychotic states mentioned above, could be of use in research aiming to identify susceptibility to mental illness.

In this study, we explore the electrophysiological signature of perception of the hollow-mask illusion. We use the P300 and P600 event-related potentials (ERPs), occurring between 300 to 600 ms and 600 to 800 ms after stimulus onset, respectively, to explore the timeline of the hollow-mask illusion. These ERPs have been previously shown to be significantly reduced in amplitude in schizophrenia patients who respond to the hollow-mask illusion experiment compared to controls (Dima et al., 2011). The P300 occurrence involves attentional engagement responsible for memory functioning and stimulus evaluation, with familiar stimuli activating context- and familiarity-related temporo-parietal top–down control. The P600 has been traditionally thought to reflect any linguistic processes; however, studies have also implicated it to similar processes as the P300, in that it is triggered when a subject encounters an “improbable” stimulus (Coulson, King & Kutas, 1998). Since ungrammatical sentences are relatively rare in natural speech, a P600 may not be simply a linguistic response but rather an effect of the subject’s “surprise” upon encountering an unexpected stimulus (Coulson et al., 1998). Higher amplitudes in P300 and P600 are believed to be associated with stimulus novelty and significance, which are modulated by late perceptual processes, such as remembering and attention (Stelmack, Houlihan & McGarry-Roberts, 1993), and lower amplitude in P300 and P600 during perceiving concave faces was assumed to be a result in late perceptual processing dysregulation in patients with schizophrenia (Dima et al., 2011). Electroencephalography (EEG) studies have associated these late positive event-related components with frontal, temporal and parietal scalp distribution (Polich, 2007).

In this study, we investigate the P300 and P600 ERPs during the presentation of hollow-mask stimuli in healthy participants in relation to their individual differences in neuroticism and anxiety self-report measures. Neuroticism, from the five-factor model (McCrae & Costa, 1992), is of particular interest in psychiatry, as it reflects dysregulation of the emotional equilibrium, anxiety proneness, and susceptibility to stress (Hettema, Neale, Myers, Prescott & Kendler, 2006). As such, high scores in neuroticism are also associated with comorbidity of schizophrenia and anxiety disorders (Caspi, Houts, Belsky & Goldman-mellor, 2015; Khan, Jacobson, Gardner, Prescott & Kendler, 2005). A meta-analysis on the five-factor model has found that higher levels of neuroticism in individuals with schizophrenia come in conjunction with lower levels of extroversion, with large effect sizes, and lower openness, agreeableness and conscientiousness, but with more moderate effect sizes (Ohi et al., 2016). Additionally, there is evidence that schizophrenia appears to be co-occurring along with anxiety disorders (Muller, Koen, Seedat, Emsley & Stein, 2004; Temmingh & Stein, 2015). There is a complex relationship between anxiety and positive symptom expression in psychotic states. Trait and state anxiety (STAI; Spielberger et al., 1983) can be seen as predictors of paranoia in psychosis spectrum disorders (Freeman & Fowler, 2009; Cowles & Hogg, 2019), whereas state anxiety mediates intrusive thoughts in hallucinating schizophrenia patients (Bortolon, Capdevielle & Raffard, 2015). Previous reports have shown that trait anxiety is significantly associated with positive psychotic symptoms and auditory hallucinations in schizophrenia patients, making it a potential causal factor for the disorder (Guillem et al., 2005). However, when the authors controlled for state anxiety, the correlation with hallucinations became non-significant whilst a significant relationship with bizarre delusions was revealed (Guillem et al., 2005). Hence, state anxiety concealed the delusion-trait anxiety relationship and revealed that delusions mediate the relationship between hallucinations and trait anxiety (Guillem et al., 2005). Accordingly, there is a complex relationship of self-reported neuroticism and anxiety self-reports with psychopathology, and particularly with psychosis which is known to be implicated with abnormal ERPs during the presentation of hollow-mask stimuli.

Given the predisposition of elevated neuroticism and anxiety levels with general psychopathology and psychosis, we hypothesised that self-reported neuroticism and anxiety levels will have an effect on the late positive ERPs that reflect high-order cognitive processes and are generated by hollow-mask stimuli. Furthermore, based on the presented evidence, it is expected that our sample consisting of healthy participants will be susceptible to the illusion behaviourally and show no difference in P300 and P600 amplitude for the concave and convex face stimuli. However, we expect to find an effect of neuroticism and anxiety scores on the amplitude of the ERPs generated by the concave and convex faces.

## 1 Methods

### 1.1 Participants

Neuroticism data using the Neuroticism Extroversion Openness Personality Inventory-Revised (NEO PI-R) (McCrae & Costa, 1992) were collected from 94 participants from 18 to 59 years of age *(M* = 26.21*, SD* = 11.67*)*, of which 72 were female and 22 were male (Table 1). Exclusion criteria consisted of: (i) lifetime history of mental disorder or substance use, (ii) reported head injury or medical disorder, and (iii) intake of prescribed psychiatric medication. Participants provided written informed consent prior to their inclusion and the study was approved by the Psychology Department Ethics Committee of City, University of London. Participants were self-referred from study advertisements throughout the university and word-of-mouth recommendation. All participants completed the mini-international neuropsychiatric interview (MINI) (Sheehan et al., 1998).

**Table 1.** Demographic and questionnaire data in the entire sample (*N* = 94)

Demographic data	
Gender (male: female)	22:72
Age in years, *M* (*SD*)	26.21 (11.67)
Age range	18–59
NEO PI-R scores	
Neuroticism, *M* (*SD*)	97.74 (22.76)
Neuroticism range	46–162

NEO PI-R: Neuroticism Extroversion Openness Personality Inventory-Revised; STAI: State–Trait Anxiety Inventory.

Upon analysis of the neuroticism trait, 20 out of the 94 subjects were invited to participate in the hollow-mask illusion EEG paradigm. The selection was designed to include a normal distribution of neuroticism scores (Table 2). Those who took part in the EEG session first completed the State–Trait Anxiety Inventory (STAI) to assess state and trait measures of anxiety (Spielberger, Gorsuch, Lushene, Vagg & Jacobs, 1983). Following this, they participated in the hollow-mask illusion experiment while their brain activity was being recorded with EEG.

**Table 2.** Demographic, questionnaire, and behavioural data in the EEG sample (*N* = 20)

Demographic data	
Gender (male: female)	3:17
Age in years, *M* (*SD*)	25.70 (8.96)
Age range	18–59
NEO PI-R scores	
Neuroticism, *M* (*SD*)	103.60 (25.77)
Neuroticism range	46–142
STAI scores	
State anxiety, *M* (*SD*)	33.75 (9.42)
Trait anxiety, *M* (*SD*)	43.75 (9.95)
Correct responses to face stimuli (% correct), *M (SD)*	
Concave	16% (23%)
Convex	86% (18%)
Flat	69% (26%)
Response time for face stimuli in ms, *M* (*SD*)	
Concave	4117.68 (.36)
Convex	3181.45 (1202.75)
Flat	3400.43 (1072.18)

EEG: Electroencephalography; NEO PI-R: Neuroticism Extroversion Openness Personality Inventory-Revised; STAI: State–Trait Anxiety Inventory.

### 1.2 The NEO PI-R and STAI self-report questionnaires

The NEO PI-R is a 240-item self-report questionnaire, grouped in five meta-factors, each having six distinct facets. It is used to measure five broad dimensions of personality traits in adults, namely neuroticism, extroversion, openness, agreeableness, and conscientiousness resulting from the scores of their corresponding facets (McCrae & Costa, 1992). Responses for each item have a five-point scale ranging from strongly disagree to strongly agree. In our analysis, we only focus on neuroticism.

The STAI is a 40-item self-report questionnaire and was devised by Spielberger et al. (1983). It is used to measure state and trait measures of anxiety, which result from its two forms, Y-1 and Y-2 respectively, each consisting of 20 items. Responses for each item have a four-point scale: “not at all”, “somewhat”, “moderately so”, and “very much so”.

### 1.3 Stimuli and design

Participants were included in the study only if their vision was normal or corrected to normal, had normal colour vision, and had functional stereoscopic vision. Stereoscopic vision was tested using the TNO test, designed by the by the Institute for Perception, Netherlands Organisation for Applied Scientific Research (Lameris Ootech BV, Utrecht, Netherlands; (http://www.ootech.nl/). Furthermore, prior to the hollow-mask illusion experiment, participants took a further test to evaluate if they have functional stereopsis. They viewed images of 15 geometric shapes of three possible surface curvatures (concave, convex and flat) and were asked to respond according to their perception. They were included in the study only if they got all 15 correct.

Subsequently, the hollow-mask illusion experiment was conducted to test the perception of binocular depth inversion (Dima et al., 2011). During this experiment, participants observed images of upright or upside-down faces (real faces) on a computer monitor with the aid of a Wheatstone mirror stereoscope (Wheatstone, 1838, 1852). They were told that the curvature (depth perception) of the faces will vary and were instructed to press one of three keys according to their perception of the depth of the image: “Concave” when a face was perceived as 3D going inwards to the screen, “Convex” when it was going outwards (like normal faces), and “Flat” when they perceived the face as 2D.

In order to create the impression of 3D, each eye was presented with a photo of the same face taken from two angles that corresponded to the views of the left and right eyes. The participant was able to perceive a 3D face that was fused in the middle of the screen while looking through the stereoscope. The effect of binocular depth inversion was generated by swapping the images for the right and left eyes; this swapping has the effect of creating a stereo pair with opposite binocular disparities to those of the convex face and produce, a concave face. Flat (2D) faces were produced by presenting images from the same angle (i.e. the same photo) to both eyes.

Participants performed 12 blocks, each containing 24 stimuli (12 upright and 12 upside-down), one-third concave, one-third convex, and the other third flat, presented in a random order. This led to six different conditions (upright convex, upright concave, upright flat; upside-down convex, upside-down concave, upside-down flat), resulting in 288 images per participant during the course of a complete experimental session. Each stimulus was presented until participants responded. A tone was heard 1.2 s after stimulus onset that signalled to participants that they were free to make a response according to their depth perception of the stimulus. The inter-stimulus interval, following response, was 0.5 s and the whole session lasted for an average of 45 min, including breaks. In all subsequent analyses, only upright faces are included.

### 1.4 EEG acquisition and ERP analysis

The EEG signal was recorded using a 64-channel, BrainVision BrainAmp series amplifier (Brain Products, Herrsching, Germany) with a 1000 Hz sampling rate. The data were recorded with respect to FCz electrode reference. Ocular activity was recorded with an electrode placed underneath the left eye. Pre-processing was conducted in BrainVision Analyser (Brain Products, Herrsching, Germany) and the statistical analysis of the ERP was conducted in the Statistical Package for the Social Sciences software (SPSS 23, Armonk, NY: IBM Corp).

**Figure 1.**
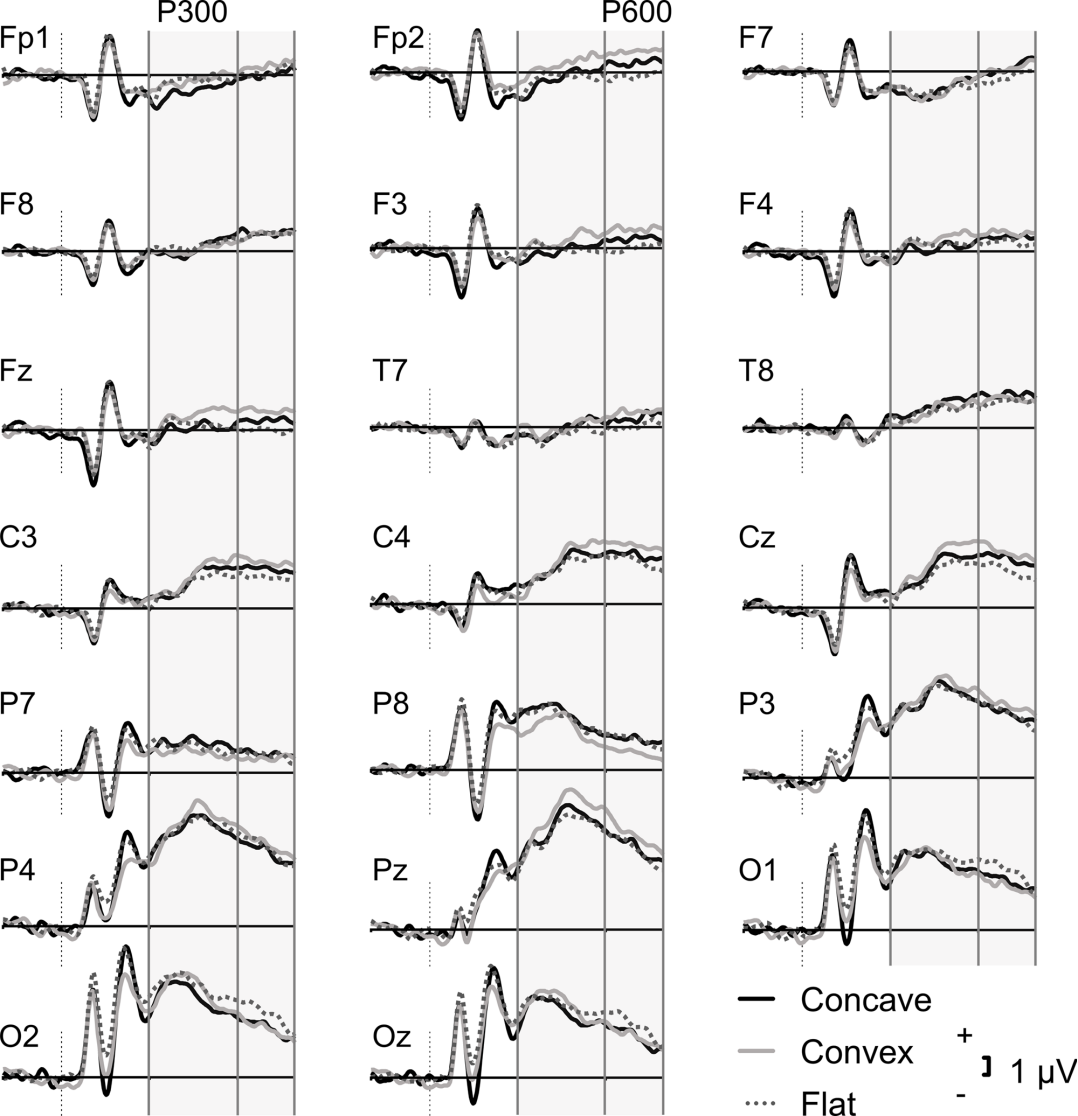
Grand average ERP waveforms for 3D normal faces, 3D inverted faces and flat faces in the whole sample (*N* = 20) in the 20 ROI electrodes.

**Figure 2.**
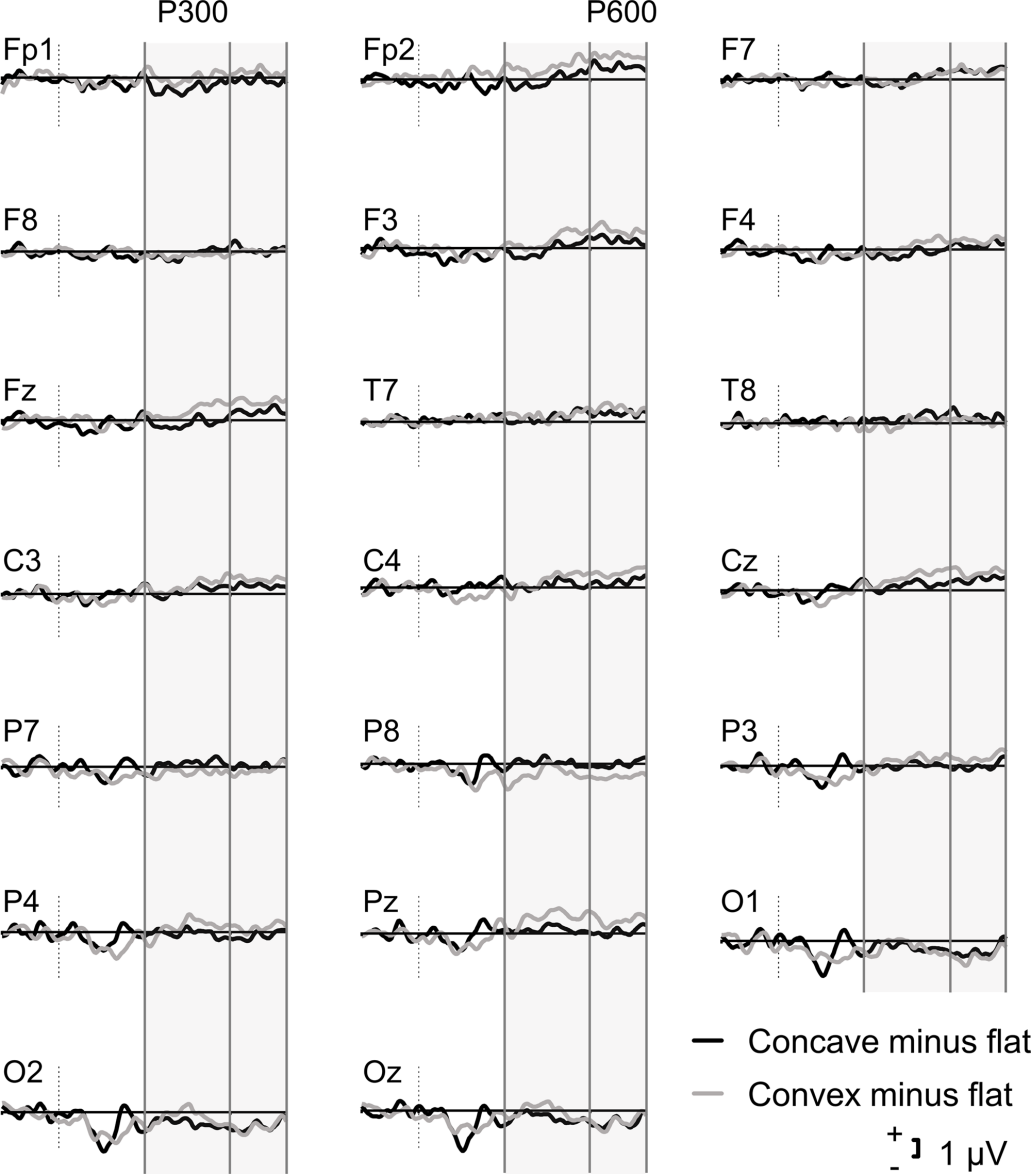
Grand average ERP difference waves for 3D normal faces minus flat faces and 3D inverted faces minus flat faces in the whole sample (*N* = 20) in the 20 ROI electrodes.

Pre-processing steps are described in their order of application. First, all EEG channels were individually inspected for high-frequency noise artefacts and slow drift. Those which were noisy throughout the whole EEG session were topographically interpolated by spherical splines. Subsequently, EEG data were down-sampled to 250 Hz and a high-pass filtered with a cut-off frequency of 0.5 Hz was applied. An automatic ocular correction was performed with the independent component analysis in BrainVision Analyser. Following re-referencing to TP9 and TP10 electrodes, data were segmented from 200 ms prior to 1000 ms after stimulus presentation for each condition. A low-pass filter of 30 Hz was applied followed by automatic artefact rejection which excluded segments with a slope of 100 µV/ms, min–max difference of 200 µV in a 200 ms interval and low activity of 0.5 μV in a 100 ms interval. Baseline correction was applied using the 200 ms interval preceding the stimulus and averaging was performed across each condition (convex, flat, concave). Averaging included all trials per condition (≈48), as opposed to only focussing on accurate-only responses, since concave faces were almost impossible to identify correctly (Table 2). For illustration purposes, a high cut-off filter of 20 Hz was applied to the grand average ERPs in Figure 1 and 2.

### 1.5 Statistical analysis

After pre-processing, the grand average data were extracted from BrainVision Analyser and were taken into SPSS for statistical analysis. The mean amplitudes of the ERPs were separately analysed for the 300–600 ms (P300) and the 600–800 ms (P600) time windows after stimulus onset. The waveforms of the flat faces were used as a baseline to calculate the difference waves for the concave and convex faces. As discussed by Luck (2014), this process can be used to eliminate identical components between separate conditions and isolate those that differ. Difference waves of “concave minus flat” and “convex minus flat” were used to moderate for face processing-related activity and allow comparison of the different 3D features between depth-inverted and depth non-inverted conditions. This led to the creation of two new variables that were used in the analysis as the “condition” factor with two levels: (i) the mean amplitude of the concave minus flat (Concave) and (ii) the mean amplitude of the convex minus flat (Convex).

Electrodes from five separate regions-of-interest (ROIs) were included in the analyses: frontal (Fp1–Fp2–F7–F8–F3–F4–Fz), central (C3–C4–Cz), temporal (T7–T8–P7–P8), parietal (P3–P4–Pz), and occipital (O1–O2–Oz). The electrode ROIs were chosen to correspond with those used in Dima et al. (2011). Repeated measures analyses of variance (RM ANOVA) with factors electrode ROI × condition as well as RM ANCOVA, with a .01 alpha-level (α) after Bonferroni correction (.05/5: for the five electrode ROIs) were run in SPSS to examine the main effects the condition factor (Concave vs Convex) and its interactions with neuroticism, state and trait anxiety scores as covariates. Additionally, Pearson’s correlations were used to test the associations of the anxiety and neuroticism measures.

## 2 Results

### 2.1 Personality and anxiety scores

Mean scores of neuroticism, state and trait anxiety, and their standard deviations are shown in Table 2 for the 20 participants in the EEG session. Neuroticism, state and trait anxiety scores were distributed normally based on Shapiro–Wilk tests (*p* > .05). Correlation analysis showed that neuroticism had a positive correlation with trait anxiety (*r* = .673, *p* = .001) and state anxiety (*r* = .480, *p* = .032). Also, state and trait anxiety were positively correlated with each other (*r* = .447, *p* = .048). Age or sex did not have effect on neuroticism or on state and trait anxiety (*p* ≥ .265).

### 2.2 Behavioural data

Table 2 shows the percentages of correct classification of the stimuli in the three conditions (concave, convex and flat), as well as their corresponding response times (RT). Correct responses for the concave faces, as expected, accounted only for 16% (*SD* = ±23%) of the trials, far below the convex (*M* = 87%; *SD* = ± 17%) and flat (*M* = 66%; *SD* = ±28%) faces. Correct responses for convex faces were significantly higher than for concave faces (*t*_*19*_ = 11.330, *p* < .001) but not for flat ones (*t*_*19*_ = 2.390, *p* = .270). Whereas, correct responses for flat faces were significantly higher than the concave responses (*t*_*19*_ = 9.120, *p* < .001). Concave faces were misclassified equally as flat (*M* = 47%, *SD* = 21%) or convex (*M* = 39%, *SD* = 21%), *t*_*19*_ = 1.000, *p* = .329. RT for concave faces were significantly longer than convex RT (*t*_*19*_ = 3.094, *p* = .006), but not for the flat ones (*t*_*19*_ = 1.939, *p* = .067). Also, flat RT was not significantly different from convex RT (*t*_*19*_ = 1.911, *p* = .071).

RT and correct responses for the three types of stimuli (concave, convex, and flat faces) did not significantly correlate with neuroticism or either measure of anxiety.

### 2.3 ERP results

#### 2.3.1 Main effects

Figure 1 illustrates the grand average ERP waveforms for the concave, convex, and flat stimuli trials in all electrode ROIs. The difference waves for the two conditions (concave minus flat; convex minus flat) are illustrated in Figure 2.

RM ANOVA revealed a significant main effect of condition (convex vs concave) in the mean amplitudes of the P300 difference wave in the temporal area (*F*_1,19_ = 6.267, *p* = .022), that did not survive Bonferroni correction. For the remaining four electrode groups, namely the frontal, central, parietal, and occipital, no significant main effects were detected in the P300 or P600 time windows (*p* > .05).

#### 2.3.2 Neuroticism

Neuroticism did not interact significantly with condition (concave/convex) in either P300 or P600 time window in any of the five electrode ROIs that were tested (*p* ≥ .099).

#### 2.3.3 State anxiety

RM ANCOVA for the P300 ERP showed significant interactions for the condition × covariate in the central (*F*_1,18_ = 10.044, *p* = .005, *η*_*p*_^*2*^ = .358) and parietal (*F*_1,18_ = 9.243, *p* = .007, *η*_*p*_^*2*^ = .339) ROIs. A significant interaction was found in the frontal ROI (*F*_1,18_ = 4.820, *p* =.041, *η*_*p*_^*2*^ = .211) that did not survive multiple correction.

In the P600, a condition × state anxiety interaction that was significant for multiple comparisons was observed in the parietal ROI (*F*_1,18_ = 13.270, *p* = .002, *η*_*p*_^*2*^ = .424). For the temporal (*F*_1,18_ = 4.772, *p* = .042, *η*_*p*_^*2*^ = .210), central (*F*_1,18_ = 4.712, *p* = .044, *η*_*p*_^*2*^ = .207), and occipital (*F*_1,18_ = 5.023, *p* = .037, *η*_*p*_^*2*^ = .218) ROIs, significant interactions were found; however, they did not survive Bonferroni correction.

**Figure 3.**
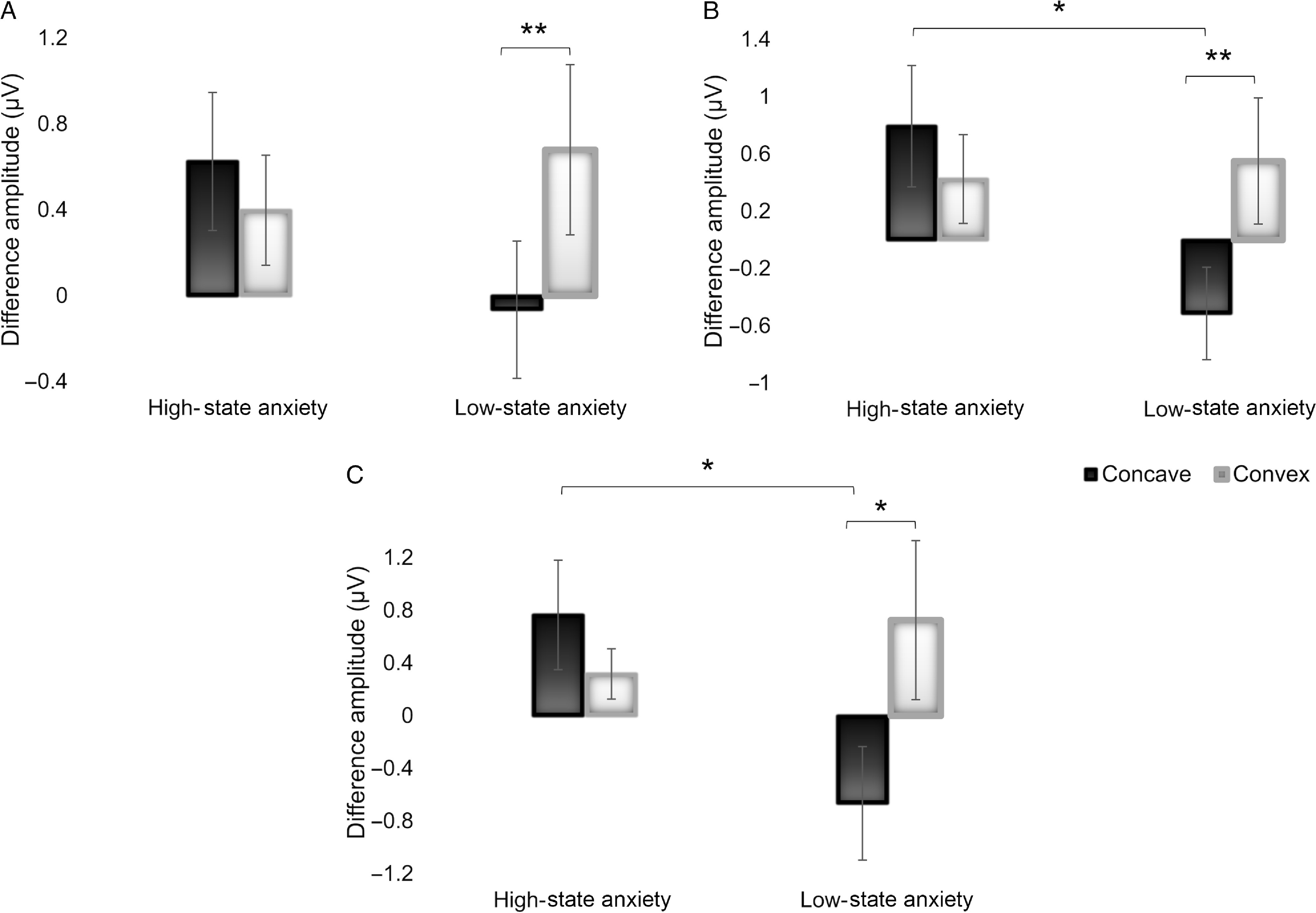
Mean difference amplitude (μV) of concave and convex ERPs minus flat for high-state anxiety and low-state anxiety groups by median split, in A) the P300 central, B) P300 parietal and C) P600 parietal time windows; * *p* < .05; ** *p* < .01 for between- and within-group comparisons.

Subsequently, significant interactions of the continuous state anxiety covariate were explored by creating a categorical variable for state anxiety. Participants were separated according to their state anxiety scores by median split (*Mdn* = 33) into two groups of high-state and low-state anxiety (Bishop, Jenkins & Lawrence, 2007). Hence, 9 participants were included in the high-state anxiety group and 11 in the low-state anxiety group. There was no significant difference for behavioural scores between the two groups, except for correct responses to convex faces with low-state anxiety participants identifying them more correctly (*p* = .031). Subsequently, the mean difference amplitudes and standard errors were calculated for each electrode ROI that showed a significant interaction with the 3D condition, namely the central and parietal for the P300 time window and the parietal for the P600 (Figure 3). Post-hoc independent *t*-tests showed high- vs low-state anxious participants had significantly higher amplitudes for concave faces in the parietal ROI for the P300 (*t*_18_ = 2.498, *p* = .022, *Cohen’s d* = 1.123) and P600 (*t*_18_ = 2.359, *p* = .030, *Cohen’s d* = 1.060), but not in the central P300 (*t*_18_ = 1.507, *p* = .149, *Cohen’s d* = .678). Concurrently, paired-sample *t*-tests revealed significant differences between the 3D conditions only in the low-state anxiety group participants. The difference amplitude in the concave condition was significantly lower in the central and parietal P300 (*t*_10_ = 3.810, *p* = .003, *Cohen’s d* = 1.149; *t*_*10*_ = 3.527, *p* = .005, *Cohen’s d* = 1.064) and the parietal P600 (*t*_10_ = 2.818, *p* = .018, *Cohen’s d* = .850) (Figure 3).

#### 2.3.4 Trait anxiety

For the P300 time window, trait anxiety showed significant interactions with the condition factor at frontal (*F*_1,18_ = 5.310, *p* = .033, *η*_*p*_^*2*^ = .228) and central (*F*_1,18_ = 5.879, *p* = .026, *η*_*p*_^*2*^ = .246) ROIs, not surviving Bonferroni correction.

In the P600 time window, significant condition × trait anxiety interactions in frontal (*F*_1,18_ = 4.819, *p* = .042, *η*_*p*_^*2*^ = .211) and central (*F*_1,18_ = 6.732 and *p* = .018, *η*_*p*_^*2*^ = .272) ROIs did not survive Bonferroni correction.

## 3 Discussion

To our knowledge, this is the first study to assess neuroticism, state and trait anxiety in ERPs during the hollow-mask illusion. There are three key findings from our study. First, as expected, (i) controls rarely perceive concave 3D faces as they fail to correctly categorise them as such and (ii) there were no main significant effects of condition (convex/concave) in the P300 and P600 time windows (Dima et al., 2011). Second, there was no interaction of neuroticism or trait anxiety and concave/convex condition in the P300 and P600 time windows while viewing the hollow-mask illusion, not supporting our initial hypothesis. Third, there was a significant interaction of state anxiety and with the condition (concave/convex) in the P300 time window at central and parietal electrodes, and in the P600 time window at parietal ones.

The interaction effect between concave/convex faces and state anxiety revealed that the difference of amplitudes of the concave condition for the P300 ERP was significantly lower compared to the convex, only in the low-state anxiety group (Figure 3). This was the case despite the absence of conscious perception of concave stimuli in both groups. The P300 component is traditionally associated with the detection of an expected but unpredictable target in a series of stimuli, like the oddball task. The P300 is thought to be composed of two subcomponents, the “novelty” component (P300a), a large fronto-central positive wave elicited by novel stimuli that mainly reflects involuntary attention shifts to changes in the environment and the “target” component (P300b), more relevant to our study (Polich, 2007). The “target” component is generated in posterior-parietal brain areas and reflects memory access processes that are activated by stimuli that require an evaluation or input (Giraudet, St-Louis, Scannella & Causse, 2015).

In the low-state anxiety group, the concave condition elicited significantly smaller P300 amplitude than the convex condition. Although participants rarely reported concave faces as concave and misclassified them as convex or flat, they were highly accurate in correctly reporting both convex and flat faces. A recent study that tested different types of expectations (target stimuli could either confirm or disconfirm passive or active expectations) has shown that expected stimuli like the convex faces used in our study, could be related to larger P300 amplitudes (Król & El-Deredy, 2015). The authors argued that conscious expectations can indirectly affect expectancy and thus have an opposite direction effect of automatically formed expectations. Additionally, Kok (1988) showed that the P300 tends to be smaller when the stimulus does not match the active representation (template mismatch). In line with this, the P300 in our study was smaller when the concave face activated a convex face representation, compared to when a stimulus is less probable (probability mismatch) in the low-state anxiety group. There has also been evidence that the P300 is smaller for difficult tasks, especially when uncertainty is greater and a resolution much harder to reach (Polich, 1987); this uncertainty becomes evident by the longer RTs for concave faces. Our results also question the prevailing theory that the P300 amplitude increases with greater mental resource allocation (Polich & Kok, 1995) and increased informational content of the stimulus, reflecting extraction and utilisation of information (Gratton et al., 1990).

The high-state anxiety group compared to the low-state anxiety group showed a stronger P300 in response to the concave condition. Only a few studies have investigated the P300 in patients with anxiety disorders. In a study using the auditory oddball stimuli, the results showed not only clear differences between subjects who suffered from anxiety and controls but also showed opposite results between anxious patients and anxious controls; while anxious patients compared to controls showed a decreased P300, the group of anxious controls compared to anxious patients showed an increase of the P300 (Boudarene & Timsit-Berthier, 1997). Anxious participants have also been shown to display higher emotional sensitivity and enhanced P300 peak amplitude to negative emotional words compared to non-anxious participants (De Pascalis, Strippoli, Riccardi & Vergari, 2004; Naumann, Bartussek, Diedrich & Laufer, 1992). An early P300 subcomponent (P315) was also larger in patients having an anxiety disorder alone when compared to depressed patients with or without an anxiety disorder and controls when performing an auditory oddball task; whereas a late P300 subcomponent (P400) was larger in patients having comorbidity of anxiety and depressive disorders than in the controls and depressed patients (Bruder et al., 2002). Another study looking at source characteristics of the P300b showed an anxiety-related pattern of hyperactive ventral attention networks for the anxiety group, indicating increased stimulus-driven attention to task-relevant stimuli (Li et al., 2015). In a recent meta-analysis of ERPs in post-traumatic stress disorder, results demonstrated that seven studies (out of eight) showed increased P300 responses to trauma related or aversive stimuli in the post-traumatic stress disorder group compared to the control group (Javanbakht, Liberzon, Amirsadri, Gjini & Boutros, 2011). In terms of P300, the present study disclosed a greater sensitivity to concave faces in anxious subjects, with a higher P300 amplitude indicating a greater effort investment for these subjects (Brocke, Tasche & Beauducel, 1997). There might be increased attentional resource allocation in anxious subjects to the concave faces showing sensitisation (sensitisation is a learning process in which repeated exposure of a stimulus results in the progressive amplification of a response) to stimuli that are not consciously correctly reported as concave. Our results therefore add support to the notion of impaired attentional resources in anxious participants leading to shifting more resources – hyperarousal – to stimuli that are mismatches (concave faces) compared to stimuli that are expected (convex faces).

The same pattern of interaction between state anxiety and condition can be seen in the P600 time window at parietal electrodes. The P600 is a centro-parietal late positive EPR that has been associated with syntactic operations such as successful retrieval and recollection (Kaan & Swaab, 2003). The P600 amplitude is known to increase with words being consciously remembered (Smith, 1993), as well as remembering not only the words but also the context of encoding (Wilding, Doyle & Rugg, 1995; Wilding & Rugg, 1996). Furthermore, it is larger for deeply encoded items implying sensitivity to the levels of processing manipulation (Rugg et al., 1998). The language specificity of the P600 has been challenged with studies showing salience and probability of stimulus occurrence affecting P600 amplitude (Coulson et al., 1998; Gunter, Stowe & Mulder, 1997). In the Coulson et al. study (1998), both ungrammatical and improbable stimuli elicited larger P600 amplitude, while in the Gunter et al. (1997) study stimulus probability and sentence complexity had similar influence on the P600. Thus, it is not surprising that in the low-state anxiety group, convex faces elicit a strong P600 while the concave faces that are rarely correctly classified and are misrepresented as convex or flat faces elicit a much smaller P600. However, the opposite effect is seen in the high-state anxiety group implying the same mechanism we see in the P300 time window. Studies have demonstrated significantly higher amplitudes of the P600 in an obsessive–compulsive disorder patient group compared to controls in a working memory paradigm (Papageorgiou & Rabavilas, 2003) as well as in a selective attention task (Towey et al., 1994). Thus, the pattern of the results obtained in the current study suggests that the high-state anxiety group demonstrated impairments in the later stages of information processing as they are reflected by the stronger P600 elicited while viewing the concave face that involve or affect parietal brain areas.

Accordingly, it appears that the late perceptual ERPs of low-state anxiety participants are more like the group as a whole (i.e., the response to the convex stimuli is higher than the concave (when flat is subtracted)). Increased sensitisation to novel stimuli (concave faces) at the higher end of the state anxiety levels interferes with the allocation of attention to the expected ones (convex faces). Fucci, Abdoun & Lutz (2019) have demonstrated increased amplitude in an earlier auditory component (P2) corresponding to standard stimuli compared to deviant ones under safe but not under threatening conditions (Fucci et al., 2019). Likewise, under anxiety-inducing conditions the authors observed increased frontal P2 response to the deviant stimuli (Fucci et al., 2019). Similarly, increased P300 response was also observed in a sample of behaviourally inhibited adolescents with a history of an anxiety disorder compared to adolescents with no such history (Reeb-Sutherland et al., 2009). Our results suggest a dimensional effect of state anxiety on the attentional processes that underlie the hollow-mask illusion, the low-state anxiety individuals do not need to allocate as much attention to mismatch stimuli (concave faces) while individuals experiencing high anxiety orient excessive attention to it.

Even though the interaction between trait anxiety and neural correlates of the hollow-mask stimulus did not survive Bonferroni correction, it showed the same pattern as state anxiety. Anxiety in general has been related to hypervigilance and attentional biases (in terms of intrinsic negativity by selecting threatening stimuli instead of neutral or positive stimuli) (Eysenck, 1997); however, the effects of state vs trait on these processes are not well established. Trait anxiety influences state anxiety levels and is considered a stable personality characteristic, whereas state anxiety is more of a transitory response to a situation (Meijer, 2001). There have been theories that posit that the two subtypes influence cognition differently; state anxiety decreases a person’s threshold for threat stimuli and this occurs more frequently in participants with a high score on trait anxiety (Mathews & Mackintosh, 1998; Williams, Watts, MacLeod & Mathews, 1997). It seems that state anxiety is more sensitive to electrophysiological changes related to the hollow-mask illusion paradigm compared to trait anxiety, although both subtypes influence it in similar ways. It is, however, important to acknowledge that, in our sample, state anxiety levels are moderate. Future studies should recruit participants representing a wider range of state anxiety scores to include anxiety measures at the higher end of the spectrum. This could be better addressed by incorporating a bigger sample size, despite our large effect sizes, which would ensure the inclusion of a more substantial number of individuals to capture the whole gamut of state and trait anxiety measures. Finally, it would be important that future studies should control for effects of physiological arousal by measuring heart rate and cortisol levels, although one previous study did not find these to correlate with state anxiety (Jansen, Gispen-de Wied & Kahn, 2000).

With this study, we intended to explore the relationship between the electrophysiological response to hollow-mask stimuli and traits of personality and anxiety states in controls, to indirectly inform us as to whether certain individuals are more vulnerable to psychopathology. We expected to find a relationship between neuroticism and the ERPs generated by the concave and convex faces due to neuroticism’s high occurrence in disorders that interact with the hollow-mask illusion, though this was not the case. Neuroticism has been indicated as an important risk factor for psychiatric traits including anxiety disorders (Hettema et al., 2006) and schizophrenia (Hayes, Osborn, Lewis, Dalman & Lundin, 2017; Van Os & Jones, 2001). A recent meta-analysis has found that a neurotic personality remained a significant risk factor for common mental disorders including anxiety, it was only identified as a vulnerability factor for psychotic disorders (Jeronimus, Kotov, Riese & Ormel, 2016). Research has yet to clarify whether the associations between neurotic traits and psychiatric disorders indicate whether neurotic personality characteristics are a causal factor or a consequence of psychiatric illnesses or both. In our study, we did however find significant correlations between neuroticism and state/trait anxiety. Neuroticism, also known as emotional instability (McCrae & Costa, 1997), and anxiety are closely related measures. This becomes apparent as anxiety itself makes up one of the six facets of neuroticism in the five-factor model of McCrae and Costa (1992). Trait anxiety positively correlated with neuroticism in a sample of patients with panic disorder (Foot & Koszycki, 2004). In a different study, high-state anxiety was associated with higher neuroticism scores in a healthy sample (Bonsaksen et al., 2019). In turn, state and trait anxiety was found to intercorrelate in a sample of patients with schizophrenia (Guillem, Pampoulova, Stip, Lalonde & Todorov, 2005) and schizophrenia patients tend to score higher in STAI measures than controls (Jansen et al., 2000). While state anxiety is thought more of as an effect of psychosis (Guillem et al., 2005) and has been demonstrated to be a predictor of state-paranoia (Cowles & Hogg, 2019), trait and state anxiety was shown to be related with positive symptoms of schizophrenia, such as bizarre delusions and auditory hallucinations (Guillem et al., 2005). Importantly, both anxiety measures affect cognitive control and attentional processes in controls (Pacheco-Unguetti, Acosta, Callejas & Lupiáñez, 2010). Hence, the interaction of state anxiety with the hollow-mask ERPs could explain an indirect relationship of self-report measures and proneness to mental illness. Our paper serves as a stepping-stone to understanding psychiatric disorders and future research should parse out whether high- and low-state anxiety in different disorders, and especially in schizophrenia, alters the results found here.

In summary, our study points to a potential relationship between ERPs in the hollow-mask illusion and state anxiety. The most robust findings include a significant interaction of state anxiety with 3D condition in the P300 time window at central and parietal electrodes, and in the P600 time window at parietal ones. The high-state anxiety group shows disproportionally big P300 and P600 amplitudes to concave faces implying impaired late information processing in this group. Finally, anxious participants have impaired attentional resources by transferring more resources to the concave faces that are stimuli mismatches to our memory representations compared to convex faces that are expected.
